# Using discriminative vector machine model with 2DPCA to predict interactions among proteins

**DOI:** 10.1186/s12859-019-3268-5

**Published:** 2019-12-24

**Authors:** Zhengwei Li, Ru Nie, Zhuhong You, Chen Cao, Jiashu Li

**Affiliations:** 1 School of Computer Science and Technology, China University of Mining and Technology, Xuzhou, 221116 China; 2 Mine Digitization Engineering Research Center of Ministry of Education, China University of Mining and Technology, Xuzhou, 221116 China; 3 Institute of Machine Learning and Systems Biology, College of Electronics and Information Engineering, Tongji University, Shanghai, 201804 China; 4 KUNPAND Communications (Kunshan) Co., Ltd., Suzhou, 215300 China; 5 Xinjiang Technical Institute of Physics and Chemistry, Chinese Academy of Science, Urumqi, 830011 China; 6 Departments of Biochemistry & Molecular Biology and Medical Genetics, Alberta Children’s Hospital Research Institute, University of Calgary, Calgary, AB T2N 4N1 Canada

## Abstract

**Background:**

The interactions among proteins act as crucial roles in most cellular processes. Despite enormous effort put for identifying protein-protein interactions (PPIs) from a large number of organisms, existing firsthand biological experimental methods are high cost, low efficiency, and high false-positive rate. The application of in silico methods opens new doors for predicting interactions among proteins, and has been attracted a great deal of attention in the last decades.

**Results:**

Here we present a novelty computational model with the adoption of our proposed Discriminative Vector Machine (DVM) model and a 2-Dimensional Principal Component Analysis (2DPCA) descriptor to identify candidate PPIs only based on protein sequences. To be more specific, a 2DPCA descriptor is employed to capture discriminative feature information from Position-Specific Scoring Matrix (PSSM) of amino acid sequences by the tool of PSI-BLAST. Then, a robust and powerful DVM classifier is employed to infer PPIs. When applied on both gold benchmark datasets of *Yeast* and *H. pylori*, our model obtained mean prediction accuracies as high as of 97.06 and 92.89%, respectively, which demonstrates a noticeable improvement than some state-of-the-art methods. Moreover, we constructed Support Vector Machines (SVM) based predictive model and made comparison it with our model on *Human* benchmark dataset. In addition, to further demonstrate the predictive reliability of our proposed method, we also carried out extensive experiments for identifying cross-species PPIs on five other species datasets.

**Conclusions:**

All the experimental results indicate that our method is very effective for identifying potential PPIs and could serve as a practical approach to aid bioexperiment in proteomics research.

## Introduction

The analysis of Protein-Protein Interactions (PPIs) is a matter of cardinal significance to clinical studies, which may promote researchers valuable understanding of the internal mechanisms of biological processes and the pathogenesis of human complex diseases at the molecular level. With the rapid pace of biological experimental techniques for detecting large-scale protein interactions from different species, such as TAP [1], Y2H [2], MS-PCI [3] and protein chips [4], etc., Huge amounts of PPI-related data have been collected into many publically available databases since several decades [5, 6]. However, such biological experiments for predicting PPIs are generally costly, complicated and time-consuming. Moreover, those results produced by the methods tend to be a high ratio of both false positive and false negative [7, 8]. So the rapid and low-cost computational methods are usually adopted as a useful supplement for PPI detection.

So far, a number of innovative in silico approaches have been developed for predicting the interactions among proteins based on different kinds of data, such as protein structure [9], phylogenetic profiles [10], genomic fusion events [11], etc. However, all these methods required prior domain knowledge that limits their further application. On the other hand, owing to a large amount of protein sequence data being collected, many investigators have engaged in developing protein sequence-based computational approaches for identification of PPIs, and previous works indicate that the unique feature information embedded in protein amino acid sequences may be enough detecting PPIs [12–17]. For example, Shen et al. [18] presented a novel algorithm by combining Support Vector Machines (SVM) with a conjoint triad descriptor to construct a universal model for PPI prediction only based on sequence information. When applied to predict human PPIs, it produced an accuracy of 83.90 **±** 1.29%. Najafabadi and Salavati [19] adopted naïve Bayesian networks to predict PPIs only using the information of protein coding sequences. They found that the adaptation of codon usage could lead to more than 50% increase on the evaluation metrics of sensitivity and precision. Guo et al. [13] employed auto covariance descriptor for predict PPIs from non-continuous amino acid sequences and obtained promising prediction results. This method took full advantage use of neighbor effect of residues in the sequences. You et al. [20] proposed an improved prediction approach for PPI recognition by means of rotation forest ensemble classifier and amino acid substitution matrix. When applied to the dataset of *Saccharomyces cerevisiae*, its prediction accuracy and sensitivity arrived at 93.74 and 90.05%, respectively. Although many previous methods have achieved good results for PPIs prediction, there has still room for improvement.

This article is a further expansion of our previous works [21, 22]. In this work, we presented a novel in silico method for predicting interactions among proteins from protein amino acid sequences by means of Discriminative Vector Machine (DVM) model and 2-Dimensional Principal Component Analysis (2DPCA) descriptor. The main improvement of the method lies in the introduction of a highly effective feature representation method from protein evolutionary information to characterize protein sequence and the adoption our newly developed DVM classifier [21, 23]. More specifically, for a given protein amino acid sequence with length *L*, it would be transformed into an *L* × 20 Position-Specific Scoring Matrix (PSSM) by means of the Position Specific Iterated BLAST (PSI-BLAST) tool [24] to capture evolutionary information in the protein amino acid sequence. After multiplication between PSSMs and its transposition, a 20 × 20 confusion matrix was obtained accordingly. To acquire highly representative information and speed up the extraction of feature vector, we adopted a computationally efficient 2DPCA descriptor to capture highly differentiated information embedded in the matrix and achieved a 60-dimensional feature vector. Then, we concatenated two feature vectors corresponding to two different protein molecules in a specific protein pair into a 120-dimensional feature vector. Finally, we applied our DVM model to perform the prediction of PPIs. The achieved results demonstrate our approach is trustworthy for predicting interactions among proteins.

## Results and discussion

### Assessment of prediction performance

**Fig. 1:**
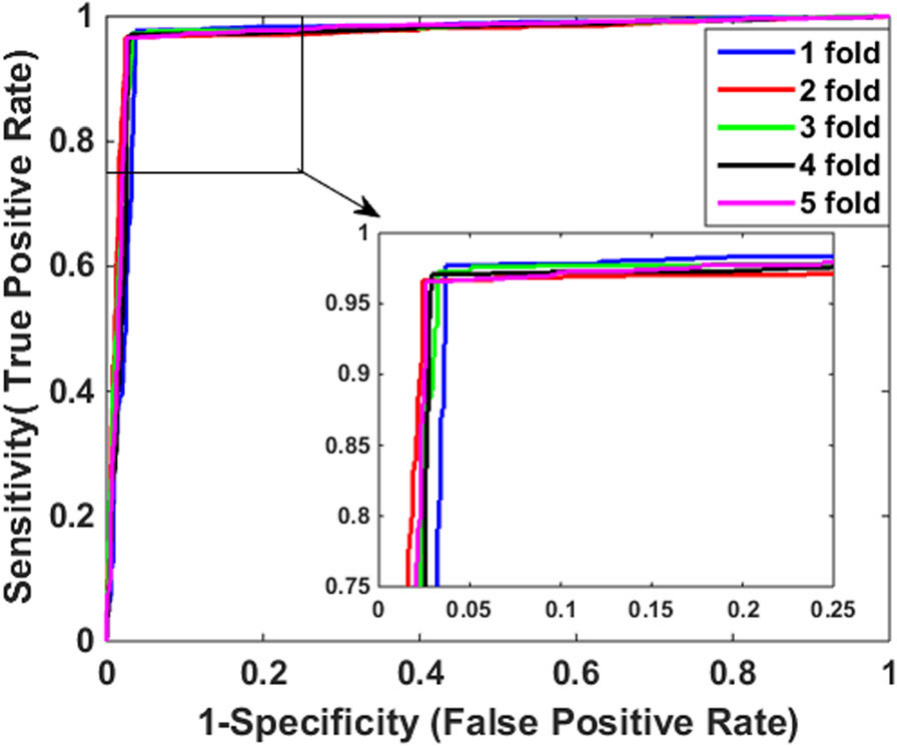
ROC curves of our model through 5-fold cross-validation based on *Yeast* dataset

**Fig. 2:**
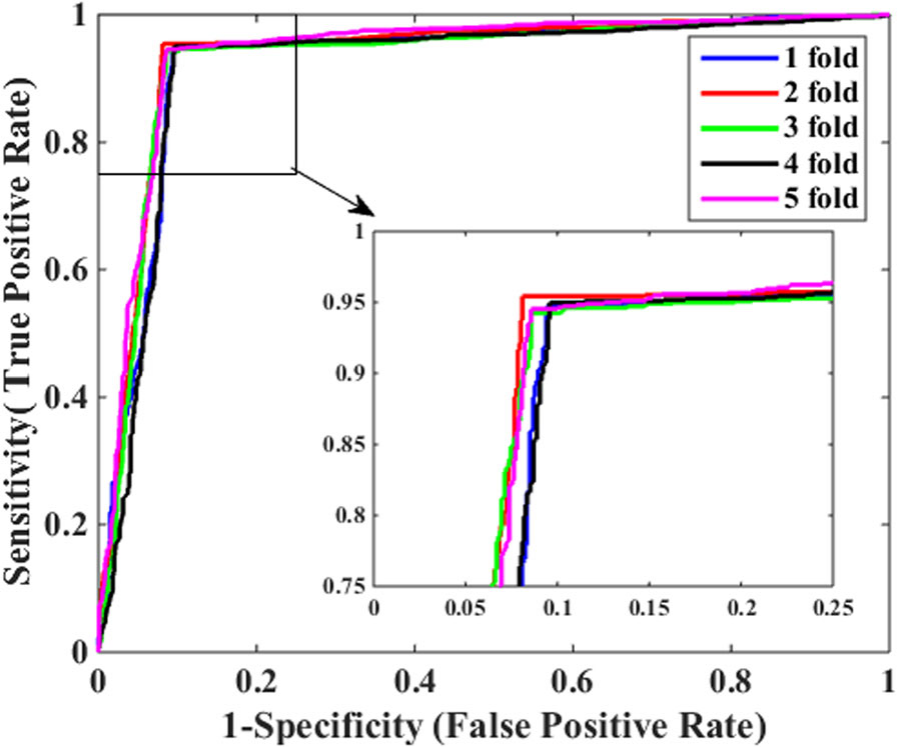
ROC curves of our model through 5-fold cross-validation based on *H. pylori* dataset

In order to avoid over fitting of predictive method and make it more reliable, 5-fold cross-validation was employed in this work. The verified dataset was permutated randomly at first and then partitioned into five parts in roughly equal size, four parts of which were used for training predictive model, and the rest part for test. In order to reduce experimental error and ensure reliability of experimental results, we repeated such permutation and partition process five times, and therefore corresponding five training sets and five test sets were generated accordingly. That is to say, we performed 5-fold cross-validation five times and the mean value of corresponding evaluation metrics were calculated as the final validation results. To be fair, all parameters of the proposed model among different processes kept the same value. The predictive results performed by combining 2DPCA descriptor with DVM classifier on *Yeast* and Helicobacter pylori (*H. pylori)* datasets are illustrated in Tables 1 and 2, respectively. It can be observed From Table 1 that our proposed approach achieves excellent performance on the dataset of *Yeast*. The mean value of accuracy (Acc), sensitivity (Sen), precision (Pre) and MCC reaches 97.06, 96.97, 96.89% and 0.9412, respectively. Similarly, when applied to *H. pylori*, just as listed in Table 2, the achieved results by our proposed method are of Acc ≥ 92.89%, Sen ≥ 90.78%, Pre ≥ 94.79% and MCC ≥ 0.8566. Besides, it can be seen from Tables 1 and 2 that their corresponding standard deviations are very low on the two datasets. The maximum value of their standard deviations on the *Yeast* dataset is only 0.38%, while the corresponding values of standard deviations on *H. pylori* dataset are as low as 0.39, 0.38, 0.46 and 0.35%, respectively. The receiver operating characteristic (ROC) curves of 5-fold cross-validation based on these datasets are shown in Fig. 1 and Fig. 2, respectively. In those two figures, the vertical axis indicates sensitivity while the horizontal axis denotes 1-sepecificity.

**Table 1:** Predictive results of 5-fold cross-validation performed by our model on *Yeast* dataset

Test set	Acc (%)	Sen (%)	Pre (%)	MCC
1	97.05	96.55	97.13	0.9410
2	97.14	97.22	96.37	0.9428
3	97.00	96.63	97.25	0.9401
4	97.09	97.18	97.09	0.9419
5	97.01	97.27	96.59	0.9402
Average	97.06 ± 0.06	96.97 ± 0.35	96.89 ± 0.38	0.9412 ± 0.0012

**Table 2:** Predictive results of our model through 5-fold cross-validation on *H. pylori* dataset

Test set	Acc (%)	Sen (%)	Pre (%)	MCC
1	92.62	90.76	94.77	0.8533
2	93.56	91.27	95.44	0.8609
3	92.76	90.80	94.23	0.8556
4	92.62	90.21	94.99	0.8537
5	92.90	90.85	94.53	0.8596
Average	92.89 ± 0.39	90.78 ± 0.38	94.79 ± 0.46	0.8566 ± 0.0035

From experimental results in Tables 1 and 2, it can be concluded that our prediction model is practically feasible for predicting interactions among proteins. We attribute its outstanding performance to the feature representation and adoption of DVM classification algorithm. In our proposed method, PSSM not only captured the location and topological information for protein amino acid sequence but also fully dug up corresponding evolutionary information. In addition, the advantage of 2DPCA to PCA rests with the former is more efficient in evaluating covariance matrix, as it can decrease the intermediate matrix transformation and improve the speed of feature extraction.

### Comparisons with SVM-based prediction model

To further verify the PPI-identification performance of our model, a SVM-based predictive model was constructed to recognize PPIs on *Human* dataset, and then the predictive results between DVM and SVM were compared accordingly. The LIBSVM tool we employed here was gotten from www.csie.ntu.edu.tw/~cjlin/libsvm. For fairness concerning, the two prediction models used same feature selection techniques. In the experiment, we selected the popular radial basis function as kernel function of SVM. Then, its two super parameters (kernel width parameter *γ*, regularization parameter *C*) were optimized by general grid search strategy and their values were finally tuned to 0.3 and 0.5, respectively.

**Fig. 3:**
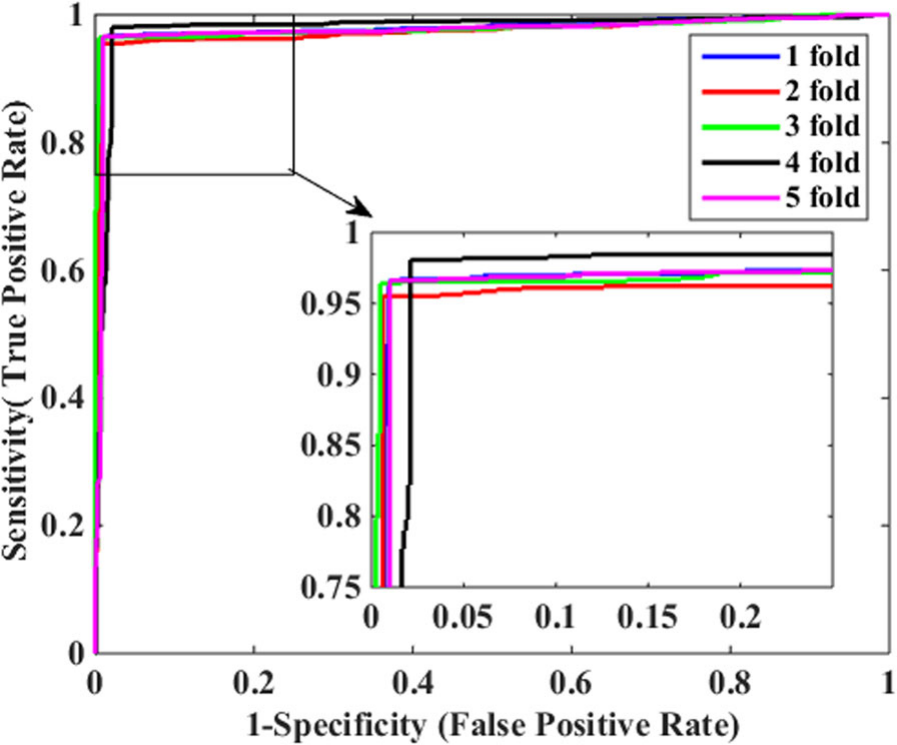
ROC curves of 5-fold cross-validation performed by DVM-based model on *Human* dataset

**Fig. 4:**
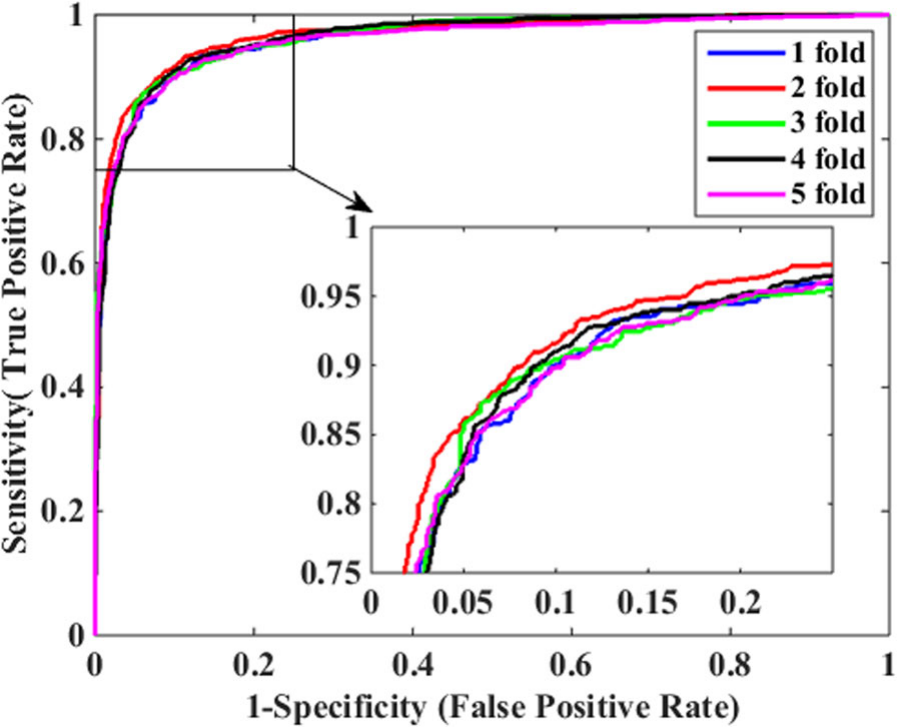
ROC curves of 5-fold cross-validation performed by SVM-based model on *Human* dataset

Table 3 illustrates the prediction results of 5-fold cross-validation over the two methods based on *Human* dataset. When using the DVM-based predictive model to identify PPIs, we obtained excellent experimental results with the mean Acc, Sen, Pre and MCC of 97.62, 97.71, 96.63% and 0.9445, respectively. In contrast, the SVM-based predictive model got inferior results with lower mean Acc, Sen, Pre and MCC of 93.20, 92.60, 92.90% and 0.8740, respectively, which indicates that DVM is superior to SVM for detecting potential interactions among proteins. Additionally, it can be seen clearly from Table 3 that DVM is more stable than SVM as the former produced smaller standard deviations for the above four evaluation indexes overall. Specifically, SVM produced standard deviations of Acc, Sen, Pre and MCC up to 0.43, 1.41, 1.18% and 0.0082, obviously higher than the corresponding values of 0.38, 0.28, 0.92% and 0.0045 by DVM. In addition, Figs. 3 and 4 illustrate the ROC curves through 5-fold cross-validation performed by DVM and SVM respectively and so we could easily observe that AUC (area under an ROC curve) values produced by DVM are visibly greater than those of SVM.

**Table 3:** Predictive results of 5-fold cross-validation performed by the two models on *Human* dataset

Model	Test set	Acc (%)	Sen (%)	Pre (%)	MCC
DVM	1	97.86	98.06	96.57	0.9473
	2	97.43	97.37	95.50	0.9393
	3	97.04	97.73	96.41	0.9401
	4	97.98	97.89	98.07	0.9495
	5	97.80	97.51	96.61	0.9462
	Average	97.62 ± 0.38	97.71 ± 0.28	96.63 ± 0.92	0.9445 ± 0.0045
SVM	1	93.79	93.40	93.52	0.8855
	2	92.69	94.06	91.15	0.8642
	3	93.42	91.44	92.57	0.8780
	4	92.93	90.78	94.33	0.8688
	5	93.18	93.30	92.95	0.8736
	Average	93.20 ± 0.43	92.60 ± 1.41	92.90 ± 1.18	0.8740 ± 0.0082

From above validation results, we can assume that DVM is more stable and effective than SVM in detecting potential interactions among proteins. There are two fundamental explanations for this phenomenon. (1) The utilization of multiple techniques, such as manifold regularization, M-estimator and kNNs, eliminates the infaust influence of kernel function to meet Mercer condition and decreases the impact of isolated points. (2) Although the number of parameters (*β*, γ, and *θ*) of DVM is more than that of SVM, these parameters have little effect on the prediction power of DVM as long as they are set in the appropriate range. In conclusion, we have reason to believe that DVM is much more suitable than SVM for PPI prediction in term of the above feature representation.

### Performance on independent dataset

Despite the exciting performance of our method in detecting interactions among proteins on the three benchmark datasets including *Yeast*, *H. pylori* and *Human* datasets, we here still made further analyses to verify our method on four well-known independent datasets (*E. coli, C. elegans, H. sapien*, *M. musculus*). In this study, we treated the all samples of *Yeast* dataset as training data and those ones coming from the other four independent datasets as test data. The feature extraction followed the same process as before. When our proposed method was applied to predicting candidate interactions among proteins for the four species, we obtained the mean values of Acc varying from 86.31 to 92.65 as listed in Table 4. The achieved results demonstrate that *Yeast* protein might possess similar functional interaction mechanism with the other four different species and using only protein sequence data could still be enough to identify potential PPIs for other species. Besides, it also indicates that the generalization ability of our proposed model is powerful.

**Table 4:** Predictive results of our proposed model on four independent datasets

Species	Test pairs	Acc(%)
*E. coli*	6954	86.31
*C.elegans*	4013	92.65
*H.sapien*	1406	91.64
*M.musculus*	312	87.72

### Comparisons with other previous models

To date, a lot of in silico methods have been developed for detecting PPIs. To further verify the predictive power of our proposed model, we also compared it with some well-known previous models based on two benchmark datasets, namely *Yeast* and *H. pylori*. Tables 5 gives the corresponding comparisons of 5-fold cross-validation of different models based on *Yeast* dataset. Just as shown in Table 5, the mean Acc values performed by other models based on *Yeast* dataset varied from 75.08% until 93.92%, but our model got the maximum value of 97.06%. Equally, the values of Sen, Pre and MCC obtained by our prediction model were also higher than those values by other previous models. Furthermore, the lowest standard deviation 0.0012 indicates our model is more stable and robust than other models. Owing to an ensemble learning model is often superior to a single classifier, although the model proposed by Wong etc. occupies the minimum standard deviation in all models, our predictive model is still very competitive in silico method for predicting potential PPIs.

**Table 5:** Predictive results of 5-fold cross-validation performed by different models on *Yeast* dataset

Model	Test set	Acc (%)	Sen (%)	Pre (%)	MCC
Guo [13]	ACC	89.33 ± 2.67	89.93 ± 3.68	88.87 ± 6.16	N/A
	AC	87.36 ± 1.38	87.30 ± 4.68	87.82 ± 4.33	N/A
Yang [25]	Cod1	75.08 ± 1.13	75.81 ± 1.20	74.75 ± 1.23	N/A
	Cod2	80.04 ± 1.06	76.77 ± 0.69	82.17 ± 1.35	N/A
	Cod3	80.41 ± 0.47	78.14 ± 0.90	81.66 ± 0.99	N/A
	Cod4	86.15 ± 1.17	81.03 ± 1.74	90.24 ± 1.34	N/A
You [26]	EELM	87.00 ± 0.29	86.15 ± 0.43	87.59 ± 0.32	0.7736 ± 0.0044
Wong [27]	RF + PR-LPQ	93.92 ± 0.36	91.10 ± 0.31	96.45 ± 0.45	0.8856 ± 0.0063
Our method	DVM	97.06 ± 0.06	96.97 ± 0.35	96.89 ± 0.38	0.9412 ± 0.0012

In the same way, Table 6 shows the comparisons of the predictive results performed by different models on *H. pylori* dataset. Our proposed model achieved the mean Acc of 92.89%, which is better than other previous models with the highest predictive Acc of 87.50%. The same situation also exists for the metrics of Pre, Sen and MCC. All the above experimental results indicate that our model combined DVM classifier with 2DPCA descriptor has better predictive performance for PPIs when compared with some other previous models. The exciting results for the prediction of PPIs performed by our proposed model might derive from the special feature representation that could extract distinguishing information, and the employment of DVM that has been validated to be an effective classifier [23].

**Table 6:** Predictive results of 5-fold cross-validation performed by different models on *H. pylori* dataset

Model	Acc (%)	Sen (%)	Pre (%)	MCC
Nanni [15]	83.70	79.00	85.70	N/A
Nanni [28]	84.00	86.00	84.00	N/A
Nanni and Lumini [29]	86.60	88.50	85.80	N/A
You [26]	87.50	88.95	86.15	0.7813
Martin [16]	83.40	79.90	85.70	N/A
Wong [27]	89.47 ± 1.05	89.18 ± 1.42	89.63 ± 1.77	0.8100 ± 0.0167
Our model	92.89 ± 0.39	90.78 ± 0.38	94.79 ± 0.46	0.85.66 ± 0.0035

## Conclusions

Owing to the advantages of time, money, efficiency and resources, in silico methods solely utilizing protein amino acid sequences for detecting potential interactions among proteins has increasingly aroused wide spread concern in recent years. In this study, we developed a novel sequence-based in silico model for identifying potential interactions among proteins, which combines our newly developed DVM classifier with the 2DPCA descriptor on PSSM to mine the embedded discriminative information. We here adopted 5-fold cross-validation in the experiments to evaluate the predictive performance, which could reduce the over-fitting to a certain extent. When applied to the gold standard datasets, our model achieves satisfactory predictive results. Furthermore, we also compared our model with SVM-based model and other previous models. In addition, to verify the generalization power of our model, we trained our model using *Human* data set and performed the prediction of PPIs based on the other five species datasets. All the experimental results demonstrate that our model is very effective for predicting potential interactions among proteins and is reliable for assisting biological experiments about proteomics.

## Materials and methodology

### Gold standard datasets

In this work, we first evaluated our model on a benchmark PPI dataset named *Yeast,* which came from the well-known Database of Interaction Proteins (DIP), version DIP_20070219 [30]. In order to decrease the interference of fragments, we deleted those protein sequences less than 50 amino acid residues in length, and picked CD-HIT [31], a common multiple sequence alignment tool, to align protein pairs with a sequence similarity threshold of 0.4. Then, we finally got 5594 interacting protein pairs to be the positive samples. The construction of negative sample is of critical importance for training and assessing predictive model of PPIs. Nevertheless, it is hard to construct high-credible negative dataset as there was only a very limited knowledge at present about non-interacting proteins. Herein, to keep the balance of the whole dataset, the negative samples containing 5594 additional protein pairs were chosen randomly at different subcellular compartments according to [32]. Accordingly, the final *Yeast* dataset here contained 11,188 protein pairs in which positive and negative samples were just half of each.

To verify the performance of our approach, we also assessed it based on the other two famous PPI datasets of *Human* and *H. pylori.* The former dataset could be downloaded from the site of http://hprd.org/download. By using the same preprocessing steps as described above, we then obtained 3899 protein pairs as positive samples and selected 4262 protein pairs coming as negative samples. Therefore, the final *Human* dataset contains 8161 protein pairs in total. Using the same strategy, the final *H. pylori* dataset contains 2916 protein pairs altogether, in which positive and negative samples account for half of each [33]. All these three datasets could be viewed as gold standard datasets for PPI prediction and were usually leveraged for comparing the performance of different methods.

### 2DPCA descriptor

The 2-Dimensional Principal Component Analysis (2DPCA) descriptor developed by Yang et al. [34] was originally employed in face representation and recognition. For an *m* × *n* matrix A, a projected vector *Y* of *A* can be obtained by the following transformation.

1 $$ Y= AX $$

where *X* is an *n*-dimensional column vector. Suppose the *jth* training sample could be represented as an *m* × *n* matrix *A*_*j*_(*j* = 1, 2, …*M*), and the mean matrix of all training samples is recorded as $\overline{A}$. Therefore, the scatter matrix of all samples *G*_*t*_ can be calculated as

2 $$ {G}_t=\frac{1}{M}\sum \limits_{j=1}^M{\left({A}_j-\overline{A}\right)}^T\left({A}_j-\overline{A}\right) $$

Then the following function *J*(*X*) can be employed to evaluate the column vector *X*:

3 $$ J(X)={X}^T{G}_tX $$

This is the so-called generalized scatter criterion. The column vector *X* maximizing the criterion can be regarded as the optimal projection axis. In practice, there may exists enormous projection axis and it is not sufficient to select only on best projection axis. We herein chose some projection axes (*X*_1_, *X*_2_, …, *X*_*d*_) that are under the orthonormal constraints and need to maximize the generalized scatter criterion *J*(*X*), namely,

4 $$ \left\{\begin{array}{c}\left\{{X}_1,{X}_2,\dots, {X}_d\right\}=\arg \max J(X)\\ {}{X}_i^T{X}_j=0,i\ne j,i,j=1,2,\dots, d.\end{array}\right. $$

Actually, those projection axes, *X*_1_, *X*_2_, …, *X*_*d*_, are the orthonormal eigenvectors of *G*_*t*_ just corresponding to the top *d* biggest eigenvalues. The optimal projection vectors of 2DPCA, *X*_1_, *X*_2_, …, *X*_*d*_, were then employed to extract feature representation. For each sample matrix *A*_*i*_,

5 $$ {Y}_k={A}_i{X}_k,k=1,2,\dots, d $$

Then, we got a set of projected feature vectors, *Y*_1_, *Y*_2_, …, *Y*_*d*_, which were just the Principal Component of the sample *A*_*i*_. In particular, each principal component in 2DPCA algorithm is a column vector, while the counterpart in PCA is just a scalar. The principal component vectors obtained by 2DPCA are employed for constructing *m* × *d* matrix =[*Y*_1_, *Y*_2_, …, *Y*_*d*_], which is employed to build feature representation of the matrix *A*_*i*_.

Since 2DPCA is based on the two-dimensional matrix directly rather than one-dimensional vector, so there is no need to transform two-dimensional matrix into one-dimensional vector prior for feature representation. Therefore, 2DPCA has higher computing efficiency than PCA and it can greatly accelerate the process of feature extraction.

### DVM

With the rapid development of software and hardware techniques, a large number of machine learning algorithms have spring up over the past several decades. In this article, our newly designed DVM classifier [23] was used for detecting candidate interactions among proteins. The DVM classifier belongs to Probably Approximately Correct (PAC) learning algorithm, which can decrease the generalization error, and has good robustness. For a test sample *y*, the objective of the DVM algorithm is to seek the *k* Nearest Neighbors (kNNs) to eliminate the impact of isolated points. The collection of k nearest neighbors of *y* is denoted as *X*_*k*_ = [*x*_1_, *x*_2_, …, *x*_*k*_]. Similarly, *X*_*k*_ can also be expressed by *X*_*k*_ = [*x*_*k*, 1_, *x*_*k*, 2_, …, *x*_*k*, *c*_], where *x*_*k*, *j*_ belongs to the *jth* category. Therefore, the goal of DVM is turned into minimizing the following function:

6 $$ {}_{\beta_k}{}^{\mathit{\min}}\ \delta \left\Vert {\beta}_k\right\Vert +{\sum}_{i=1}^d\varnothing \left({\left(y-{X}_k{\beta}_k\right)}_i\right)+\gamma \sum \limits_{p=1}^k\sum \limits_{q=1}^k{w}_{pq}{\left({\beta}_k^p-{\beta}_k^q\right)}^2 $$

where *β*_*k*_ may be expressed as $\left[{\beta}_k^1,{\beta}_k^2,\dots, {\beta}_k^c\right]$ or [*β*_*k*, 1_, *β*_*k*, 2_, …, *β*_*k*, *c*_], where *β*_*k*, *i*_ is the coefficient value of the *i* th category; ‖*β*_*k*_‖ is the norm of *β*_*k*_ and we here adopted Euclidean norm in the following calculation since it could prevent over-fitting and improve the generalization ability of the model. To improve the robustness of the model, we introduced a robust regression M-estimation function ∅ that is a generalized maximum likelihood descriptor presented by Huber to evaluate the related parameters based on loss function [35]. In comparison, we finally selected the Welsch M-estimator (∅(*x*) = (1/2)(1 −  *exp* (−*x*^2^)) for decreasing error and thus those isolated points had a small impact for predictive model. The last part in Eq. (6) plays the role of manifold regularization where *w*_*pq*_ denotes the similarity degree of the *pth* and *qth* nearest neighbors of *y*. In the experiments, we adopted cosine distance as similarity measure since it pays more attention to the difference of direction between two vectors. Next, the Laplacian matrix related to similarity measure can be denoted as

7 $$ L=D-W $$

where *W* is the similarity matrix whose element is *w*_*pq*_(*p* = 1, 2, …, *k*; *q* = 1, 2, …, *k*); *D* denotes a diagonal matrix and its element *d*_*i*_ in row *i* and column *j* is the sum of *w*_*qj*_(*q* = 1, 2, …, *k*). Followed by Eq. (7), we reformulated the final part of Eq. (6) into $\gamma {\beta}_k^TL{\beta}_k$. Besides, we also built diagonal matrix *P* = diag(*p*_*i*_) whose element *p*_*i*_(*i* = 1, 2, …, *d*) is:

8 $$ {p}_i={e}^{-\frac{{\left({\left(y-{X}_k{\beta}_k\right)}_i\right)}^2}{\sigma^2}} $$

where *σ* is the kernel width that could be expressed as:

9 $$ \sigma =\sqrt{\Big(\theta \ast {\left(y-{X}_k{\beta}_k\right)}^T\ast \left(y-{X}_k{\beta}_k\right)/d} $$

where *d* denotes the dimension of *y* and *θ* represents a threshold parameter to suppress the outliers. In the experiments, we adopted 1.0 for *θ* just same as the literature [36]. Based on formulas (7), (8) and (9), the calculation for Eq. (6) could be converted to as follows:

10 $$ \mathit{\arg}{}_{\beta_k}{}^{\mathit{\min}}{\left(y-{X}_k{\beta}_k\right)}^TP\left(y-{X}_k{\beta}_k\right)+\delta {\left\Vert {\beta}_k\right\Vert}_2^2+\gamma {\beta}_k^TL{\beta}_k $$

Based on the half-quadratic regularization strategy, the solution *β*_*k*_ for Eq. (10) could be represented by:

11 $$ {\beta}_k={\left({X}_k^TP{X}_k+\delta I+\gamma L\right)}^{-1}{X}_k^T Py $$

Once the involved coefficients were determined, the test sample *u* could be predicted to be corresponding category as long as the L2 norm of ‖*u* − *X*_*ki*_*β*_*ki*_‖ possesses the global lowest value.

12 $$ {R}_i={}_{i\ }{}^{\mathit{\min}}\left\Vert u-{X}_{ki}{\beta}_{ki}\right\Vert, i=1,2,\dots, c $$

With the help of manifold regularization and Welsch M-estimator to curb the impact from those isolated points and improve the generalization ability, our newly proposed classifier DVM possesses strong generalization power and robustness. All samples in the experiments could be divided into two categories in total: interaction protein pair (category 1) and non-interaction protein pair (category 2). If the residual *R*_1_ is lower than the residual *R*_2_, we would attribute the test sample *u* to the interaction protein pair, or else non-interaction protein pair. As for the super parameters (*δ*, *γ*, *θ*) in DVM, the cost of directly searching their optimal values is very high. Fortunately, our DVM classifier is very robust and thus those parameters have little effect on the performance for our predictive model as long as they are in the corresponding wide range. Based on the above knowledge, we optimized the model via the grid-search method. At last, we selected 1E-4 and 1E-3 for *γ* and *δ* in the experiments. As mentioned earlier, threshold *θ* was set to 1.0 during the entire process of the experiments. In addition, as for large-scale dataset, DVM would take huge amount of calculation work to obtain the corresponding representative vector, and then multi-dimensional indexing and sparse representation techniques could be introduced to accelerate the computing process.

### Procedure of our proposed model

**Fig. 5:**
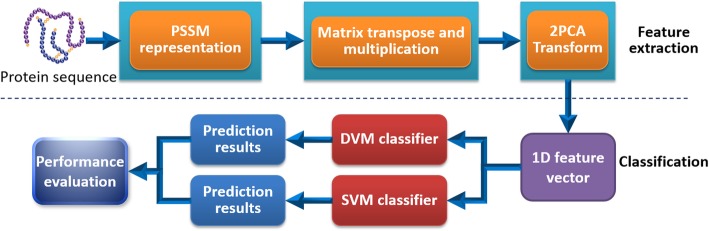
schematic flow chart of our model for predicting potential PPIs

The overall process of our predictive model could be formulated to two main steps: feature representation and classification. As the first step, feature representation itself consisted of 3 sub-steps: (1) The Position Specific Iterated BLAST (PSI-BLAST) tool [24] was employed for mining the evolutionary information from protein amino acid residue sequence and every protein molecule was expressed as a corresponding PSSM matrix. The value of e-value and iterations of PSI-BLAST were optimized for 0.001 and 3, respectively; (2) Each PSSM matrix and its transposition were multiplied and the 20 × 20 confusion matrix was obtained accordingly; (3) The application of 2DPCA descriptor, serialization and concatenation operations on the feature matrices of the corresponding protein pair were performed in order. Then, the final feature vector was formed and can be treated as the input of the subsequent classifier. Similarly, the second step of classification could be divided into two sub-steps: (1) On the basis of three benchmark datasets of *Yeast*, *H. pylori* and *Human*, our proposed model was trained with the feature representation produced by main step 1. (2) The established model was then used to predict the potential interactions among proteins on those gold datasets and the predictive performance of the model was calculated subsequently. Moreover, a predictive model based on SVM and the same feature representation was also constructed for the prediction of PPIs and the performance comparison between DVM and SVM based on *Human* dataset was performed accordingly. The main schematic flow chart of our model is shown as Fig. 5.

### Evaluation criteria

To assess the performance of our proposed model, 4 widely used evaluation indexes were employed in the experiments, such as precision (Pre), sensitivity (Sen), accuracy (Acc), and Matthews’s correlation coefficient (MCC), which could be defined by:

13 $$ \mathrm{Pre}=\frac{\mathrm{TP}}{\mathrm{TP}+\mathrm{FP}} $$

14 $$ \mathrm{Sen}=\frac{TP}{TP+ FN} $$

15 $$ \mathrm{Acc}=\frac{\mathrm{TP}+\mathrm{TN}}{\mathrm{TP}+\mathrm{FP}+\mathrm{TN}+\mathrm{FN}} $$

16 $$ \mathrm{MCC}=\frac{\left(\mathrm{TP}\times \mathrm{TN}\right)-\left(\mathrm{FP}\times \mathrm{FN}\right)}{\sqrt{\left(\mathrm{TP}+\mathrm{FN}\right)\times \left(\mathrm{TN}+\mathrm{FP}\right)\times \left(\mathrm{TP}+\mathrm{FP}\right)\times \left(\mathrm{TN}+\mathrm{FN}\right)}} $$

where TP refers to the number of physically interaction protein pairs (positive samples) identified correctly while FP represents the number of non-interaction protein pairs (negative samples) identified falsely. Equally, TN refers to the number of physically non-interaction samples identified correctly, while *FN* represents the number of physically interaction samples identified mistakenly. MCC is usually employed in machine learning for evaluating the performance of a binary classifier. Its value is located in the scale [− 1, 1], where 1 denotes a perfect identification and − 1 a misidentification. In addition, we also performed the predictive results to characterize False Positive Rate (FPR) against True Positive Rate (TPR) in term of different classification methods on several benchmark datasets. Moreover, both Receiver Operating Characteristic (ROC) curve and the Area Under an ROC curve (AUC) were employed to visually assess the predictive power for the related methods. AUC represents the probability that a positive sample is ahead of a negative one. The closer AUC is to 1.0, the higher performance of the predictive model.

## Data Availability

The datasets used and/or analysed during the current study are available from the corresponding author on reasonable request.

## Abbreviations

2DPCA: Two-Dimensional Principal Component Analysis
AUC: Area Under an ROC Curve
DVM: Discriminative Vector Machine
FP: False Positive
FPR: False Positive Rate
MCC: Matthews’s Correlation Coefficient
PPI: Protein-Protein Interaction
PSI-BLAST: Position-Specific Iterated Basic Local Alignment Search Tool
PSSM: Position-Specific Scoring Matrix
ROC: Receiver Operating Characteristic
SVM: Support Vector Machines
TP: True Positive
TPR: True Positive Rate
